# Pandora's Box

**DOI:** 10.1192/bji.2023.17

**Published:** 2023-08

## Mind the gap

The economic crisis of 2010 brought global inequalities to the surface more clearly, and over the years the gap between the rich and the poor has been increasing. The COVID pandemic has highlighted the socioeconomic disparities between and within countries and the major adverse effects these have on health and well-being for communities and individuals. This has been highlighted further with the recent major increase in the cost of living. Research has shown that levels of prosperity are relevant to brain development, and poverty can have an impact on our children, with longer-term effects on their ability to manage their economic status in adulthood. Without appropriate state policies aimed at improving people's economic status, adversity and its effects repeat from generation to generation.

## Of poverty, race and the brain

Maladaptive processes in the brain have been linked to household income more closely and to neighbourhood level characteristics such as crime more distally. Neuroimaging studies have helped to improve our understanding of the associations between environmental influences and children's brain structure.

In a recent study in the USA, researchers explored these relationships closely. They carried out a cross-sectional analysis of the baseline data-set from the Adolescent Brain and Cognitive Development (ABCD) Study, which is the largest study of brain development in children to date. They aimed to identify the socioeconomic characteristics of household and community that may be associated with potential adverse brain development outcomes. They assessed cognitive ability using the National Institute of Health Toolbox (NIH-TB) battery of tests and measures of brain cortical thickness, area and volume based on magnetic resonance imaging. Income, education, employment and housing quality data were obtained from the Area Deprivation Index percentile score, which ranks a neighbourhood according to the American Community Survey data (2011–2015), and also from the ABCD measures of housing, unemployment, education and income.

They recruited 9000 ‘typically developing’ children aged 9 to 10 years from local schools at 21 sites across the USA: 55% of the children were White, 13% were Black and 20% were Hispanic, with the boys slightly outnumbering the girls. Most of the children from the lowest-income families or resident in impoverished neighbourhoods were Black or Hispanic. NIH-TB cognitive measures were negatively associated with household and neighbourhood socioeconomic characteristics. Very importantly, though, differences in cognitive scores between Black or Hispanic children and other racial groups were mitigated by higher household income, indicating that these were related to economic and not racial status. The authors concluded that neighbourhood socioeconomic characteristics are associated with racial differences in pre-adolescent brain outcomes. They also noted that household income mediates racial differences more strongly than neighbourhood-level socioeconomic indicators.

Policy makers need to be aware and take action to decrease socioeconomic disparities that may lead to better cognitive outcomes to everybody's benefit

Isaiah A, Ernst TM, Liang H. et al. Associations between socioeconomic gradients and racial disparities in preadolescent brain outcomes. Pediatr Res [Epub ahead of print] 1 Dec 2022. Available from: 10.1038/s41390-022-02399-9.PMC1023267536456690

## Poverty, stress and the hippocampus

Another study from the USA demonstrates the relationship between low socioeconomic status and brain development, examining whether cost of living and the generosity of a state's social safety net for low-income families can moderate the association between family income, hippocampal volume and mental health. Socioeconomic status in families and communities is known to be associated with repeated exposure to stressful events such as conflict, separation and violence. Chronic stress raises cortisol levels and has a toxic effect on the hippocampus, impairing its ability to regenerate neuronal cells (neoneurogenesis) and leading to a decrease in its volume; this affects mental health.

The researchers, as in the study above, used data from the ABCD study of children in the USA. They extracted data from over 10,000 male and female children aged 9–11 years across 17 states. They found that lower income was associated with smaller hippocampal volume and higher internalising psychopathology. The associations were stronger in states where the cost of living was higher. However, they also found that in states where more generous income cash benefits were available for low-income families, the decrease in hippocampal volume related to socioeconomic disparities was reduced by 34%, bringing it close to that seen in the states with the lowest cost of living. Similar changes were also seen in psychopathology. It can be concluded that anti-poverty policies are of value in addressing the relationship of low income with brain development and mental health; policy makers should take note.

Weissman DG, Hatzenbuehler ML, Cikara M, Barch DM, McLaughlin KA. State level macro-economic factors moderate the association of low income with brain structure and mental health in U.S. children. Nat Commun 2023; 14: 2085.3713088010.1038/s41467-023-37778-1PMC10154403

## Brain development and future financial success

In this unequal consumerist world, with the ever-increasing complexity of electronic technology and the financial landscape, good cognitive ability is essential to our economic survival. Researchers from the University of Colorado, USA, and Maastricht University in The Netherlands explored the possible relationship between childhood cognitive ability and adult financial well-being.

It is known from previous research that there is a positive association between cognitive ability and financial well-being. In this study, the authors investigated the prospective effects of childhood cognitive ability on debt worth and subjective financial well-being in adulthood. They used a unique set of longitudinal data, the British Cohort Study, which followed 13 000 individuals from birth in 1970 to the present, and measured cognitive ability at age 10 as well as adult financial variables over a period of 35 years. They state that cognitive ability in childhood allows for an accurate representation of cognitive ability in later years, as this is relatively stable over the life course. They assessed financial well-being using a variety of measures including debt, household savings and investments, and subjective financial well-being. They also took into consideration childhood covariates such as the mother's and father's education and family income at age 10, as well as adult covariates such as income household size and marital status.

Notably, they found that both those of low and high cognitive ability had the lowest total debt probability, whereas those with average levels of cognitive ability had the highest debt. This non-linear relationship between cognitive ability and wealth indicators was more noticeable at the extreme ends of the cognitive ability distribution, with the majority of people showing a linear trend. The authors concluded that their data showed a linear trend in the relationship between cognitive ability and financial stress, and that their results suggest that cognitive ability has an important role in financial decision-making. However, they observed that both of these may depend on access to financial products and opportunities. They do stress that their results do not imply that individuals with lower cognitive abilities cannot achieve financial well-being. They also note that other factors such as financial literacy and risk attitudes need to be also explored in order to achieve a more complete understanding of the links between these variables.

Gladstone J, Barrett JAM. Understanding the functional form of the relationship between childhood cognitive ability and adult financial well-being. PLoS One 2023; 18(6): e0285199.3728532910.1371/journal.pone.0285199PMC10246798

## The flu, nose/throat, brain and sickness link

We know that respiratory infections such as influenza can be debilitating, and that neural responses to the infection are responsible for our sickness behaviours, i.e. fever, lethargy, aches and pains, and anorexia, as well as mood changes. The neuronal response may be also responsible for the adaptive physiological mechanisms that promote recovery. Research evidence links sickness status during infection with neuro-immunological changes. Various cytokines and direct pathogen factors such as toxins and others are released and can be detected by neurons.

In a recent study, researchers developed a mouse model aiming to characterise the neuronal mechanisms involved in influenza-induced sickness behaviour. They infected mice intranasally with influenza A virus and observed for sickness responses over the following 10–20 days. As expected, there was a reduction in food and water intake, decreased activity and reduced animal survival. The higher the virus inoculation, the more severe were the symptoms. There were also increases in prostaglandin (PGE2) levels in plasma and respiratory tissues. Using genetic tools, the investigators identified a small population of PGE2-detecting glossopharyngeal sensory neurons (petrosal GABRA1 neurons) that were essential for influenza-induced sickness behaviour in mice. Ablating these neurons eliminated influenza-induced sickness behaviour and improved survival. Genetically guided anatomical mapping showed that petrosal GABRA1 neurons project to the nasopharyngeal mucosa, with their axons extending also to the brainstem. These findings show the presence of a primary airway-to-brain sensory pathway, which, by detecting locally released prostaglandins, mediates a systemic sickness response to respiratory viral infection, making us take to our bed.

Bin NR, Prescott SL, Horio N, Wang Y, Chiu IM, Liberles SD. An airway-to-brain sensory pathway mediates influenza-induced sickness. Nature 2023; 615: 660–7.3689023710.1038/s41586-023-05796-0PMC10033449

## Keep dancing

By 2050, one in six people in the world will be over 65 years old, with those aged 85 and over showing the fastest projected increase. The likelihood of emerging physical and cognitive problems increases with age. Maintaining good health is not only the individual's concern (maintaining independence and well-being) but is also a priority for public health services.

The benefits of exercise for physical and mental health are well established, and physical activity is recommended. Unfortunately, as we grow older, we become less interested in physical activity, to our detriment. The Active Lives Adult Survey for the years 2015 to 2019, carried out in England, showed that 29% of those aged 65 to 74 years and 47% of those over 75 years old were inactive, in that they spent less than 30 min per week on moderate activity. The old were 50% less interested in increasing their physical activity compared with those in their 50s.

Physical inactivity is one of the main risk factors for diseases and mortality. But what can we do to motivate us to get off the couch and get active? Dance has been promoted as a motivator, being an activity for both mind and body, and it is believed to contribute to older adults’ physical, intellectual and social development.

A recent study examined both the feasibility of a weekly programme over a period of 1 year and its efficacy in improving physical activity levels and well-being. The researchers used a mixed-methods intervention design, recruiting adults over 55 years of age from local community groups in Yorkshire, UK. Each weekly session involved mixed-genre dance for 60 min. They used self-report measures of minutes per week of physical activity, and well-being was assessed using the EuroQol visual analogue scale (EQVAS) at baseline and at 3, 6 and 12 months. The results were assessed using the Friedman test. The researchers also carried out a thematic analysis of qualitative data. Feasibility was assessed based on class attendance, and 685 male and female participants with a mean age of 75 years were recruited to form focus groups. Thirty-eight per cent of the participants were considered to be highly deprived as per the Index of Multiple Deprivation.

The results demonstrated significant increases in both physical activity and EQVAS well-being scores across the four time points. The qualitative analysis of data from the focus groups showed that participants regarded the dance intervention favourably because of physical benefits such as increased mobility and flexibility, as well as psychological benefit in improving mood and well-being. They also note that previous studies have shown improved social interactions, enjoyment, increased confidence, and improvements in physical and subjective health. They conclude that dance is an acceptable way of increasing physical activity in older adults from economically diverse communities, across a wide age range from 55 to 97 years. Time to get off that couch and go dancing!

Britten L, Pina I, Nykjaer C, Astill S. Dance on: a mixed-method study into the feasibility and effectiveness of a dance programme to increase physical activity levels and wellbeing in adults and older adults. BMC Geriatr 2023; 23: 48.3670311610.1186/s12877-022-03646-8PMC9878484

